# Relationships among workaholism, personality, obsessive beliefs, and entrepreneurial motivation

**DOI:** 10.3389/fpsyg.2024.1503580

**Published:** 2024-12-24

**Authors:** Rojin Ghasemijalal, María José Serrano-Fernández, Maria Boada-Cuerva, Beatriz Sora, Jordi Assens-Serra, Joan Boada-Grau

**Affiliations:** ^1^Department os Psychology, Universitat Rovira i Virgili (URV), Tarragona, Spain; ^2^Department of Strategy, Leadership and People, EADA Business School, Barcelona, Spain

**Keywords:** workaholism, obsessive beliefs, family security, entrepreneurial motivation, perfectionism

## Abstract

**Introduction:**

Recent studies focusing mainly on entrepreneurial motivation have identified several variables (family security, motivation, and entrepreneurial intentions) as predictors of employee creativity. This research aims to provide insights into the underlying factors that shape entrepreneurial motivation, which can be used to develop effective strategies to support and foster entrepreneurship. In this study, we examine the relationship between workaholism, personality, obsessive beliefs and entrepreneurial motivation.

**Methods:**

The study sample was comprised of 1,106 Spanish workers (48.51% men and 51.49% women) obtained through non-probability sampling.

**Results:**

Our results showed that entrepreneurship motivation is related to personality traits. Positive relationships have been found with the variable perfectionism and intolerance of uncertainty, conscientiousness, work enjoyment, and agreeableness. Perfectionism and intolerance of uncertainty are the variables most strongly related to entrepreneurial motivation.

**Discussion:**

Our study contributes to the body of literature that examines the relationships between workaholism, personality, obsessive beliefs, and entrepreneurial motivation. The practical implications suggest that entrepreneurship support programs could benefit from considering not only entrepreneurial orientation but also other variables such as perfectionism and work enjoyment.

## Introduction

Entrepreneurial motivation is a topic that has been explored in various theoretical models to gain insights into entrepreneurial behavior (Murnieks et al., 2020). Motivation, defined as the set of energetic forces that arise both from within individuals and from their environment to initiate behavior and shape its form, direction, intensity, and duration (Mitchell and Daniels, 2003). An area within this field examines the factors that drive entrepreneurs to launch, expand, and eventually exit their ventures. Studies on entrepreneurial motivation frequently focus on distinct phases of the business development process, including venture initiation, growth, and exit (Murnieks et al., 2020).

In the current socioeconomic environment, the sustainability of organizations and companies requires constant adaptation, and for an organization to innovate and create, employees need to be motivated and provided with the necessary resources, and the work environment needs to be effectively managed (Mumford and Fichtel, 2020). Therefore, this research seeks to provide critical insights into the underlying factors that shape entrepreneurial motivation, which can be used to devise effective strategies that support and foster entrepreneurship. By examining these factors, this study contributes to the existing literature on entrepreneurial motivation. It sheds light on the impact of the environment on attitudes towards entrepreneurship and the relationship variables on entrepreneurial motivation. These insights are valuable for entrepreneurs and policymakers, who can use this information to develop effective strategies that support and foster entrepreneurship.

Entrepreneurship is a widely recognized driver of economic growth, innovation, and employment (Acs et al., 2009; Carree and Thurik, 2010; Nor, 2024). Organizations such as the European Commission (2021) are committed to fostering entrepreneurial intentions and career paths. In the current socioeconomic environment, organizations and companies require constant adaptation to be sustainable (Mumford and Fichtel, 2020). Innovation necessitates financial, material, and information resources, opportunities for exploration, and enough time to pursue novel ideas and approaches (Amabile, 1997; Glaeser and Lang, 2024).

The Global Entrepreneurship Monitor report (GEM, 2013) identifies several factors associated with entrepreneurship, including the perception of opportunities, orientation, attitudes, fear of failure, and entrepreneurial motivations. The report suggests that to foster entrepreneurship, government authorities must not only focus on providing external resources, such as capital and favorable financing terms, but also analyze potential entrepreneurs’ skills, motivations, and experiences. Governments need to adopt a holistic approach to supporting entrepreneurship, which considers the development of necessary skills, attitudes, and motivations of prospective entrepreneurs, if they are to encourage the creation of new businesses and positively impact economic growth and development (GEM, 2013). Furthermore, during Covid, the evidence regarding business activity levels was mixed, with the most common pattern showing a decline followed and then an increase (GEM, 2023).

The Deci and Ryan model (Deci and Ryan, 1985), considers different types of motivation (intrinsic and extrinsic) across three distinct levels of generality. Furthermore, the model posits that motivation should be viewed from a multidimensional perspective. Thus, it is insufficient to merely differentiate between intrinsic and extrinsic motivation; instead, these constructs must be understood as existing on a continuum, where the various types of intrinsic and extrinsic motivation range from high to low levels of self-determination (Vallerand, 2000). The relationship between entrepreneurial behaviors and motivation has been established in previous research (George and Marino, 2011; Kuhn and Galloway, 2015). Kuhn and Galloway (2015) found that a combination of intrinsic and extrinsic motivations leads to higher business performance than intrinsic motivations alone. Intrinsic motivation plays a crucial role in driving the constant pursuit of improvement, as well as the search for challenges and achievements (Lumpkin and Dess, 1996, 2001). Meanwhile, innovative capacity refers to the ability to generate and apply creative ideas that result in novel and distinctive products, services, or processes (Berrone et al., 2012; Lumpkin and Dess, 1996, 2001; Zhou et al., 2005; Vij and Bedi, 2012). Additionally, the willingness to take calculated risks involves carefully assessing the potential costs and benefits before making strategic decisions (Hult et al., 2004; Covin et al., 2006). These characteristics intertwine with proactivity in identifying opportunities, which implies a future-oriented mindset and the ability to anticipate market needs and demands (Lumpkin and Dess, 2001).

However, the interaction between these constructs remains relatively underexplored in the literature (Carsrud and Brännback, 2011). Entrepreneurship requires individuals to generate valuable ideas, and creativity is considered a fundamental aspect of entrepreneurship (Baron, 1998). Entrepreneurs must be able to creatively interpret their environment to identify opportunities within their area of expertise (DeTienne and Chandler, 2004).

The support of family and friends can also significantly impact work creativity (Madjar, 2008). Individuals with high levels of self-perceived creativity are expected to have positive attitudes towards entrepreneurship, as they may view entrepreneurship as an opportunity to express their creative potential (Kolvereid, 1996; Moriano, 2005; Kautonen et al., 2013).

Moreover, self-rated creativity may act as a precursor to entrepreneurial self-efficacy, which pertains to an individual’s belief in their capacity to execute entrepreneurial duties, such as recognizing fresh business prospects, generating new products, promoting concepts or novel innovations, resolving dilemmas, managing financial resources, securing support from others, exhibiting leadership, and making effective decisions (Moriano, 2005; Wilson et al., 2007; Phipps and Prieto, 2015; van Gelderen et al., 2008). Leadership in relation to entrepreneurship has been a topic of controversy, particularly with the emergence of new factors like humble leadership, which may represent an innovative contribution to the literature on entrepreneurial motivation (D’Errico, 2019; D’Errico and Poggi, 2019).

Previous research has focused on understanding the reasons why people start a business venture and the relationship between entrepreneurial behaviors and motivation. Morris et al. (2006) suggested that the reason for initiating a business venture is a critical determinant of growth aspirations, and individuals motivated by the desire for financial gain or challenge exhibit a higher inclination towards growth. Conversely, those motivated by discrimination or self-expression may be less inclined towards development.

The objective of this research is to examine the relationships between a set of antecedents and the three dimensions of Entrepreneurial Motivation (Family Security, Independence and Autonomy, and Intrinsic Motivation).

Entrepreneurial motivation toward family security has recently gained attention as entrepreneurs seek financial stability and security for their families through entrepreneurial activities. A new hypothesis proposes that entrepreneurial motivation toward family security can be accurately related by considering certain variables. These relationship variables include workaholism, personality traits, and obsessive beliefs, which it is suggested have a significant impact on entrepreneurial motivation toward family security.

Several studies have examined the relationship between workaholism and entrepreneurship. Clark et al. (2016) discovered a positive correlation between workaholic tendencies and entrepreneurial activity, highlighting the adverse effects of workaholism, such as burnout and decreased work-life balance. Similarly, Gorgievski et al. (2010) found that high levels of work engagement, which is related to workaholism, were positively linked to entrepreneurial intentions. Research on personality traits has also demonstrated that specific personality traits are linked to higher entrepreneurial motivation levels. For instance, Zhao et al. (2010) discovered that high levels of extraversion, openness to experience, and emotional stability were positively associated with entrepreneurial motivation.

Moreover, obsessive beliefs have been identified as a potential related variable of entrepreneurial motivation. According to Tu et al. (2023), research has shown that these beliefs, described as persistent, irrational thoughts or beliefs that are difficult to control, are positively associated with entrepreneurship and innovation. The hypothesis that entrepreneurial motivation towards family security may be accurately related by considering workaholism, personality traits, and obsessive beliefs is significant as it has implications for creating effective strategies to support and promote entrepreneurship among individuals who desire to provide financial security for their families.

This hypothesis proposes that workaholism, personality traits, and obsessive beliefs are critical indicators of entrepreneurial motivation toward autonomy and independence. It suggests that by integrating these variables into the model, an individual’s level of entrepreneurial motivation regarding independence and autonomy can be forecast with great accuracy.

Prior studies have underscored the significance of understanding the motivational factors that propel individuals toward entrepreneurship. Krueger (2000) emphasized the role of cognitive factors, particularly entrepreneurial self-efficacy, in predicting entrepreneurial intentions. Workaholism, a well-known concept, and its effect on personal and professional life have been defined and measured by Spence and Robbins (1992). In their study, workaholism, personality traits, and obsessive beliefs are significant predictors of entrepreneurial motivation toward independence and autonomy. By integrating these factors into the model, an individual’s entrepreneurial motivation level can be accurately forecast, thereby having profound implications for entrepreneurship education and training programs. We, therefore, hypothesized the following.

*Hypothesis 1*. The variables Workaholism, Personality, Impulsivity, and Obsessive Beliefs are related to Family Security.

*Hypothesis 2*. The variables Workaholism, Personality, Impulsivity, and Obsessive Beliefs are related to independence and autonomy.

According to Baron (2004), entrepreneurship is crucial for driving economic growth and innovation on a global scale. To comprehend the driving force behind entrepreneurs starting and developing successful businesses, various researchers have analyzed different factors that influence entrepreneurial motivation. One of the most extensively researched aspects is intrinsic motivation, which pertains to an individual’s internal drive to pursue a specific objective or activity for personal satisfaction rather than for external rewards such as financial gain or public recognition, as noted by Cardon et al. (2009).

*Hypothesis 3*. The variables Workaholism, Personality, Impulsivity, and Obsessive Beliefs are related to intrinsic motivations.

This research will examine the impact of different variables such as workaholism, personality, and obsessive beliefs on entrepreneurial motivation and whether these variables significantly influence the development of intrinsic motivation. Based on the hypothesis, this study aims for a model that includes these variables so that intrinsic entrepreneurial motivation can be accurately related.

## Method

### Participants

The sample consisted of 1,106 Spanish employees, with a male-to-female ratio of 48.51 to 51.49%. The mean age was 42.49 years (standard deviation = 11.25). Marital status was as follows: married (60.8%), single (6.9%), divorced/separated (23.8%), and widowed (8.5%). In terms of academic qualifications, 1.4% had no academic certificate or degree, 28.5% had completed primary education, 39.1% had completed secondary education, 18.4% held a three-year university degree, 12.6% held a five-year university degree (such as engineering or architecture), and 6% had completed a master’s degree or doctorate. The sample included employees from various organizations, including multinationals (7.41%), SMEs (71.24%), cooperatives (0.92%), and public administration (20.43%).

### Instruments

To assess work addiction, we have used two different questionnaires that we believe are complementary. On one hand, the DUWAS, which refers to two factors aimed at alleviating the anxiety and guilt feelings that arise from not working: working excessively and working compulsively. On the other hand, the WOrkBAT evaluates motivational and enjoyment aspects. Its factors are *Driven*, related to work aspects such as motivation, involvement, guilt, and commitment, and *Work Enjoyment*, which refers to behaviors associated with enjoyment, fun, and having a good time while working. In addition to other questionnaires that we present below.

The Dutch Work Addiction Scale (DUWAS; Schaufeli et al., 2006) is a commonly used tool that assesses the construct of workaholism. The questionnaire has two dimensions referred to as Working Excessively (WkE) and Working Compulsively (WkC). In the extended version of the survey, the WkE scale is assessed using 13 items (e.g., “I often find myself in a hurry and racing against the clock”). In comparison, the WkC scale involves eight items (e.g., “I feel an obligation to work diligently, even when the work is not enjoyable”). The response format is a 4-point Likert scale (1 = *almost never* to 4 = *almost always*).

The Workaholism Battery (WorkBAT; Burke et al., 2002; McMillan et al., 2002; Spence and Robbins, 1992) is a validated psychometric instrument that assesses the construct of workaholism. A Spanish version of the WorkBAT was later developed by Boada-Grau et al. (2013). The WorkBAT consists of 19 items and two subscales: the Driven subscale (comprising 12 items, such as “I feel guilty when I take time off work” and the Work Enjoyment subscale containing 7 items, such as “My job is more like fun than work”). The WorkBAT utilizes a five-point Likert scale as its response format, from 1 (*Do not agree at all*) to 5 (*Agree*). The Driven and Work Enjoyment subscales possess alpha coefficients of 0.82 and 0.83, respectively, indicating a high consistency.

The Overall Personality Assessment Scale (OPERAS; Vigil-Colet et al., 2013) is a questionnaire based on the Big Five personality factors. It consists of 40 items. This theory posits that five fundamental personality traits determine human behavior: Extraversion (OP.EX) (α = 0.86; e.g., “20. I make friends easily”), Emotional Stability (OP.ES) (α = 0.86; e.g., “15. I often feel sad”), Conscientiousness (OP.CO) (α =0.77; e.g., “28. I am a perfectionist”), Agreeableness (OP.AG) (α =0.71; e.g., “29. I am often unpleasant with others”), and Openness to Experience (OP.OE) (α = 0.81; e.g., “24. I like to visit museums”). The survey participants were requested to indicate the degree to which they agreed with the depiction of their characteristics across 40 items on a 5-point scale ranging from “1. *Enormously disagree*” to “5. *Strongly agree*.” This scale provides scores unaffected by two of the best-known response biases: social desirability and acquiescence.

The Inventory of Obsessive Beliefs (ICO; Belloch et al., 2003) is a psychometric instrument developed to evaluate obsessive-compulsive tendencies among individuals. Belloch et al. (2003) proposed that the Spanish adaptation of the ICO consists of 58 items grouped into seven factors. These factors are scored using a 7-point Likert scale, where one represents “Strongly disagree,” and seven means “Strongly agree.” The present study focuses on two specific factors of the ICO, namely Perfectionism and Intolerance of Uncertainty (ICO.PE), and Excessive Responsibility (ICO.RE) and the Importance of Controlling Thoughts. The first factor comprises 14 items and has a Cronbach’s alpha coefficient of 0.86, as illustrated by item 2, “I must be the best at things that are important to me.” The second factor, Responsability and Control comprises 10 items and has a Cronbach’s alpha coefficient of 0.84, as exemplified by item 49, “I should be able to rid my mind of inadequate thoughts.”

The Impulsivity Inventory (DII; Dickman, 1990), Spanish version (Chico et al., 2003), is a psychometric tool that consists of 23 items and two subscales. The first subscale, Functional Impulsivity (IMP.F), comprises 11 items and has been found to possess a basic level of internal consistency, with a coefficient alpha of 0.77. This subscale evaluates an individual’s ability to take advantage of unexpected opportunities that require immediate action. An example item on this subscale is “4. I am good at taking advantage of unexpected opportunities, where you have to do something immediately or lose your chance.” The second subscale evaluates dysfunctional impulsivity (IMP.D), with a coefficient alpha of 0.76. This subscale assesses an individual’s tendency to act without thinking, resulting in hurried situations. An example item on this subscale is “14. Frequently, I get into hurried situations because I do not think before acting.” The Likert response options for each item are 1 (*True*) and 0 (*False*).

The Entrepreneurial Motivation Scale (EM; Robichaud and McGraw, 2008) is a psychometric instrument used to evaluate an individual’s motivation to initiate professional and business ventures. The French version of the scale consists of 17 items and four factors. In contrast, the spanish scale developed by Boada-Grau et al. (2016) has 13 items and a structure of three factors: Family Security (EM.FS) (4 items; *α* = 0.75; for example, “To be better prepared for my children”), Independence and Autonomy (EM.IA) (5 items; α = 0.84; for example, “Being able to decide what I want to do”), and Intrinsic Motivations (EM.IM) (4 things; α = 0.78; for example, “To increase the profits and sales of my business”). These three factors have been found to possess adequate reliability, as demonstrated by Boada-Grau et al. (2021). The Cronbach’s alpha coefficients relating to the three factors exhibit a high degree of internal consistency, ranging from 0.77 to 0.83. The response format utilized for the scale involved a Likert scale, which varied from “*not at all important*” (1) to “*very important*” (5).

### Procedure

The sample for this study was collected using non-probabilistic sampling, also called random-accidental selection (Kerlinger, 2001) during the years 2022 and 2023. Before collecting the data, we received permission from the company managers to conduct the research. After obtaining consent and contacting employees to participate, the scales were administered to each participant individually during their work hours. The respondents were provided with clear instructions to answer the surveys and were informed that their responses would be treated with strict confidentiality and anonymity. The data collection process was conducted at a time mutually agreed upon with each participant, typically lasting 40 min. The participation of respondents was entirely voluntary and unpaid.

### Data analysis

To begin, the Kolmogorov–Smirnov test was applied to assess the normality of the data, which indicated a good fit. Additionally, the diagrams for all the regressions were analyzed, and no issues related to homoscedasticity or excessive residuals were observed.

We used Pearson’s correlation coefficients to calculate the correlations between variables. Multiple regressions were then calculated utilizing stepwise option in SPSS 26.0. Afterward, multiple regression was performed employing the stepwise option, which added each variable to the model based on its contribution to the variance explained. There were 13 variables beloging to Driven (WbDR), Work Enjoyment (WbEN), Work Excessively (WkE), Work Compulsively (WkC), Extraversion (OP.EX), Emotional Stability (OP.ES), Conscientiousness (OP.CO), Agreeableness (OP.AG), Openness to Experience (OP.OE), Functional impulsivity (IMP.F), Dysfunctional impulsivity (IMP.D), Perfectionism and Intolerance of Uncertainty (ICO.PE) and Responsability and Control (ICO.RE) to check its relationship with the criterion variables Family Security (EM.FS), Independence and autonomy (EM.IA), Intrinsic motivation (EM.IM). This method facilitated the recognition of the variables that demonstrated the optimal explanation for the maximum variance of the three criterion variables.

## Results

### Correlation analysis

The outcomes of an investigation exploring the significant correlations among different variables are illustrated in Table 1. A total of 52 positive correlations were identified. The study found a positive correlation between the three factors of Entrepreneurship Motivation and Workaholism (DUWAS & WorkBat) and Perfectionism and Intolerance of uncertainty and Responsibility and control (ICO) and Responsibility with Family Security and Independence and autonomy.

**Table 1:** Pearson correlation matrix to examine the relationship among variables.

	1	2	3	4	5	6	7	8	9	10	11	12	13	14	15
Family security															
Independence and autonomy	0.972**														
Intrinsic motivation	0.481**	0.504**													
Driven	0.233**	0.222**	0.264**												
Work enjoyment	0.154**	0.157**	0.191**	0.377**											
Work excessively	0.178**	0.173**	0.183**	0.698**	0.305**										
Work compulsively	0.220**	0.210**	0.179**	0.778**	0.252**	0.783**									
Extraversion	0.028	0.018	0.036	0.002	0.086	−0.024	−0.010								
Emotional stability	−0.031	−0.024	0.010	−0.100*	0.105*	0.018	−0.075	0.194**							
Conscientiousness	0.128*	0.106*	0.055	0.019	0.016	−0.027	0.008	0.183**	0.200**						
Agreeableness	−0.071	−0.083	0.035	−0.007	0.038	−0.041	−0.088	0.059	0.116*	−0.020					
Openness to experience	−0.010	−0.016	0.075	0.010	0.069	−0.024	0.001	0.152^**^	−0.058	0.061	0.087				
Funct. impulsivity	0.037	0.047	0.078	−0.005	0.102*	0.038	0.014	−0.023	−0.021	−0.003	0.008	0.058			
Dysf. impulsivity	0.079	0.053	−0.025	0.152**	0.153**	0.112*	0.128*	0.015	−0.030	0.042	−0.015	0.132**	0.026		
Perfectionism	0.339**	0.339**	0.364**	0.434**	0.183**	0.324**	0.389**	0.049	−0.062	0.073	0.002	0.111*	−0.097	0.099	
Responsability	0.319**	0.306**	0.355**	0.360**	0.103*	0.294**	0.337**	0.028	−0.096	0.124*	0.025	0.143**	−0.072	0.065	0.835**

*p < 0.05, **p < 0.01.

Variables used in the research: Family Security (EM.FS), Independence and autonomy (EM.IA), Intrinsic motivation (EM.IM), Driven (WbDR), Work Enjoyment (WbEN), Work Excessively (WkE), Work Compulsively (WkC), Extraversion (OP.EX), Emotional Stability (OP.ES), Conscientiousness (OP.CO), Agreeableness (OP.AG), Openness to Experience (OP.OE), Functional impulsivity (IMP.F), Dysfunctional impulsivity (IMP.D), Perfectionism and Intolerance of Uncertainty (ICO.PE), Responsability and Control (ICO.RE).

### Multiple regression

We performed a multiple regression model to test the effects of independents variables (13) on criterion variables. In this type of regression, the first variable to be introduced into the equation will be the one with the highest correlation, whether positive or negative, with the dependent variable, followed by the next highest, and so on. This statistical approach enables dependent variables to be evaluated (Hinton et al., 2014). The analysis results, including adjusted *R*2 indices and significant beta coefficients, are presented in Tables 2–4.

**Table 2:** Summary of the models, variables, and coefficients of regression analysis (step-by-step method) for Family Security (EM.FS).

Models and variables	Models						Coefficients				
	*R*	R2	R2 adjusted	R change	F change	Sig	*B*	SE	*β*	*t*	Sig
Model 1	0.348	0.121	0.118	0.121	45.155	0.000					
Model 2	0.367	0.134	0.129	0.013	5.075	0.025					
Model 3	0.382	0.146	0.138	0.012	4.444	0.036					
ICO.PE							9.185	1.191	0.320	7.712	0.000
OP.CO							0.069	0.011	0.118	6.141	0.000
WbEN							0.040	0.018	0.110	2.297	0.022

Variables used in the model: Driven (WbDR), Work Enjoyment (WbEN), Work Excessively (WkE), Work Compulsively (WkC), Extraversion (OP.EX), Emotional Stability (OP.ES), Conscientiousness (OP.CO), Agreeableness (OP.AG), Openness to Experience (OP.OE), Functional impulsivity (IMP.F), Dysfunctional impulsivity (IMP.D), Perfectionism and Intolerance of Uncertainty (ICO.PE), Responsibility and Control (ICO.RE).

**Table 3:** Summary of the models, variables, and coefficients of regression analysis (step-by-step method) for Independence and autonomy (EM.IA).

Models and variables	Models						Coefficients				
	*R*	R2	R2 adjusted	R change	F change	Sig	*B*	SE	β	*t*	Sig
Model 1	0.347	0.120	0.118	0.120	44.694	0.000					
Model 2	0.364	0.132	0.127	0.012	4.572	0.033					
Model-3	0.378	0.143	0.135	0.011	4.109	0.043					
ICO.PE							0.086	0.014	0.325	6.231	0.000
WbEN							0.078	0.036	0.115	2.199	0.029
OP.AG							−0.045	0.022	−0.104	−2.027	0.043

Variables used in the model: Driven (WbDR), Work Enjoyment (WbEN), Work Excessively (WkE), Work Compulsively (WkC), Extraversion (OP.EX), Emotional Stability (OP.ES), Conscientiousness (OP.CO), Agreeableness (OP.AG), Openness to Experience (OP.OE), Functional impulsivity (IMP.F), Dysfunctional impulsivity (IMP.D), Perfectionism and Intolerance of Uncertainty (ICO.PE), Responsibility and Control (ICO.RE).

**Table 4:** Summary of Model-5, variables, and regression analysis coefficients (stepwise method) for Intrinsic motivation (EM.IM).

Models and variables	Models							Coefficients			
	*R*	R2	R2 adjusted	R change	F change	Sig	*B*	SE	β	*t*	Sig
Model 1	0.344	0.118	0.115	0.118	43.660	0.000					
Model-2	0.379	0.143	0.138	0.025	9.622	0.002					
ICO.PE							0.071	0.012	0.315	6.031	0.000
WbEN							0.094	0.030	0.162	3.102	0.002

Variables used in the model: Driven (WbDR), Work Enjoyment (WbEN), Work Excessively (WkE), Work Compulsively (WkC), Extraversion (OP.EX), Emotional Stability (OP.ES), Conscientiousness (OP.CO), Agreeableness (OP.AG), Openness to Experience (OP.OE), Functional impulsivity (IMP.F), Dysfunctional impulsivity (IMP.D), Perfectionism and Intolerance of Uncertainty (ICO.PE), Responsibility and Control (ICO.RE).

The analysis in Table 2 in relation to Family Security (EM.FS), shows that model 3, wich includes Perfectionism and Intolerance of Uncertainty (ICO.PE), Conscientiousness (OP.CO) Factor, and Work Enjoyment (WbEN), explain 13.8% of the variance of the criterion variable. The beta coefficient values for these variables indicate that ICO.PE (*β* = 9.185), OP.CO (*β* = 0.320), and WbEN (*β* = 0.118) are statistically significant in relationing Family Security and show a positive relationship between the variables introduced in the model.

According to Table 3, the adjusted *R*2 value for Independence and Autonomy is 0.135, and is influenced by three indicators: Perfectionism and Intolerance of Uncertainty (ICO.PE), Work Enjoyment (WbEN), and Agreeableness (OP.AG) Factor. The beta coefficient values indicate that ICO.PE (*β* = 0.325), WbEN (*β* = 0.115), and OP.AG Factor (*β* = −0.104) are statistically significant variables. The results suggest that Independence and autonomy (EM.IA) can be related through the variables Perfectionism and Intolerance of Uncertainty (ICO.PE), and Work Enjoyment (WbEN) influencing positively, and Agreeableness (OP.AG) influencing negatively.

The analysis in Table 4 in relation to Intrinsic Motivations (EM.IM), shows that model 2, wich includes Perfectionism and Intolerance of Uncertainty (ICO.PE), and Work Enjoyment (WbEN) explain 13.8% of the variance of the criterion variable. The beta coefficient values indicate that ICO.PE (β = 0.315) and WbEN (β = 0.162) are statistically significant variables. Both variables have a positive influence on Intrinsic Motivations. The results suggest that Intrinsic Motivations can be explained through the variables Perfectionism and Intolerance of Uncertainty (ICO.PE), and Work Enjoyment (WbEN).

## Discussion

The notion that family security is central to shaping entrepreneurial motivation has received growing attention in entrepreneurship research. This hypothesis posits that personal characteristics, behaviors, and dispositions are crucial factors in determining an individual’s motivation to start and operate a business, this perspective aligns with the growing body of literature aimed at understanding the drivers behind individuals choosing entrepreneurship as a career path. The existing literature, as reviewed by Benzíng et al. (2009), suggests that personal and family security, alongside economic factors, independence, and internal satisfaction, are considered vital motivators for entrepreneurs in starting new ventures (Shabbir and Gregorio, 1996; Swierczek and Thai, 2003). Concurrently, research has explored how societal changes have impacted family structure. One notable study by Bitler et al. (2004) found that these changes significantly affected family structure, decreasing rates of divorce and marriage. However, another study by Fitzgerald and Ribar (2004) found no evidence to indicate that these societal changes affected the prevalence of single-parent households.

The explanatory variables outlined in the hypothesis encompass a range of personality traits, behavioral tendencies, and dispositions that are believed to influence entrepreneurial motivation. For instance, OPERAS-measured (OPERAS; Vigil-Colet et al., 2013) extraversion and emotional stability are considered essential traits for entrepreneurs, as they can impact their capacity to engage with others and deal with stress in the face of ambiguity (Cuesta et al., 2018).

The evaluation of entrepreneurs’ motivation is crucial, as it is an aspect that impacts their behavior both before and after the start of a venture (Kuratko et al., 1997). The type and magnitude of an individual’s entrepreneurial motivation can determine the goals and aspirations of the enterprise, contributing to a spectrum of macroeconomic outcomes (Fernandez-Serrano and Romero, 2013; Fernandez et al., 2009; Hessels et al., 2008). Additionally, traits such as responsibility, Agreeableness, and openness to experience are believed to play a significant role in an individual’s capability to manage a successful business, because they influence their decision-making processes, collaboration abilities, and receptiveness to new prospects.

In conclusion, the first hypothesis, that family security plays a role in shaping entrepreneurial motivation, is supported by existing empirical evidence within the field of entrepreneurship. Cheraghi (2017) highlighted various external adverse conditions, such as unemployment, dissatisfaction with one’s current job, job loss, low-paying positions with limited upward mobility, and concerns over future family security, which can serve as push factors that attract individuals towards entrepreneurship. These findings indicate the significance of considering external factors when analyzing entrepreneurial motivation. The relationship between entrepreneurs’ motivation and the success of their enterprises is a well-recognized area of study in both developed and developing countries (Isaak, 2016). However, it is essential to recognize that this is a complex and multi-dimensional topic, and not all individuals with the relevant explanatory variables necessarily exhibit entrepreneurial motivation. Additional research is required to enhance our comprehension of the interrelationship between explanatory variables and entrepreneurial inspiration.

The second hypothesis, that independence and autonomy play a role in shaping entrepreneurial motivation, has received considerable attention in entrepreneurship research. Shane et al. (2003) argued that this is further supported by research on entrepreneurship and satisfaction, which highlights the significance of autonomy. Additionally, societal trends that favor increased self-reliance further underscore the importance of this factor. The self-determination theory and self-directed learning perspectives offer insights into how autonomy can be effectively incorporated into entrepreneurship education.

The desire for autonomy and self-direction has been proposed as a fundamental motive for individuals’ interest in working in smaller firms. This is supported by the findings of Jubari et al. (2017), who provided evidence of the importance of autonomy in entrepreneurship as a career. The need for independence has also been identified as a predictor of an individual’s suitability to an entrepreneurial role (Bhardwaj and Mittal, 2017; Vecchio, 2003), further emphasizing its significance in this area.

The concept of entrepreneurial motivation is an essential aspect of entrepreneurship research. Entrepreneurial motivation is a critical predictor of subsequent entrepreneurial behavior, and individuals willing to take calculated risks and believe in their capabilities are assumed to drive the economy. According to Scarborough (2012), entrepreneurs’ driving motivations are profit, personal growth, and self-belief, and the desire to establish an entrepreneurial entity in an environment characterized by risk and uncertainty. The discussion on entrepreneurship has focused on exploiting entrepreneurial opportunities, resulting in a lack of research on initiating the process (Carsrud and Brännback, 2011). Miller et al. (2012) argue that employees with high entrepreneurial motivation scores are more likely to consider entrepreneurship as a career option.

By exploring the impact of entrepreneurial motivation among employees working in companies on the willingness to become an entrepreneur, the study sheds light on the mismatch between entrepreneurship-promoting efforts and outcomes observed by Mahto and McDowell (2018). The study’s findings are expected to contribute to the literature on entrepreneurship and the individual identity formation process (Ashforth and Schinoff, 2016).

The third hypothesis, regarding intrinsic motivation, has garnered considerable attention in entrepreneurship research as a relationship factor of entrepreneurial conduct. Perwin (2003) explains that inherent motivation is an innate inclination towards a particular task, whereas extrinsic motivation entails receiving external rewards for engaging in a specific behavior. Investigating entrepreneurial motivation is crucial to comprehending the motivating forces behind individuals’ decisions to pursue entrepreneurship (Lee and Wong, 2004). Previous research on entrepreneurial intention (EI) has explored various factors influencing an individual’s decision to start a new venture, including personality traits, socio-demographic characteristics, and capital availability. However, the phonological approach to predicting start-up decisions has yet to be successful (Linan and Santos, 2007). In *The Theory of Planned Behavior*, Ajzen (1991) provides a more extensive framework for comprehending EI. His approach accounts for the interplay between societal and personal factors and the impact of attitudes, subjective norms, and perceived control on intention.

The key drivers of EI have been identified as DSE (Desire for Self-Employment), FSE (Fear of Self-Employment), TR (Tendency to Risk), and PG&NGS (Perceived Growth and Non-Growth Situations) (Ummah, 2009). Uncertainty is also essential in predicting self-employment intention, as individuals need knowledge and motivation to risk starting a new venture (McMullen and Shepherd, 2006). Successful entrepreneurs tend to possess specific key drivers, such as independence, achievement, internal locus of control, risk-taking ability, innovation, self-confidence, and proactivity. In total, entrepreneurial and intrinsic motivation play a significant role in determining an individual’s entrepreneurial intention (McStay, 2008).

In conclusion, the three hypotheses are partially demonstrated (Table 5), since only some explanatory variables are significant (Perfectionism and intolerance to uncertainty, Conscientiousness, Work enjoyment and Agreeableness). Existing discussions on entrepreneurship support the hypothesis that family security plays a role in shaping entrepreneurial motivation. Personal and family security, economic factors, independence, and internal satisfaction are critical motivators for individuals to start new ventures. Related variables such as extraversion, emotional stability, responsibility, Agreeableness, and openness to experience also significantly shape entrepreneurial motivation and have been linked to an individual’s success in managing a business. The desire for autonomy and self-direction has also been identified as a fundamental motive for individuals interested in entrepreneurship. The current study explored the relationship of entrepreneurship motivation on the willingness of employees to become entrepreneurs.

**Table 5:** Summary of the regression analysis for the criterion variables.

Explanatory variable	Factor 1 family security		Factor 2 independence and autonomy		otivationmntrinsic iFactor 3	
	ΔR2 corrected	*β*	ΔR2 corrected	*β*	ΔR2 corrected	*β*
Perfectionism and intolerance of uncertainty (ICO.PE)	0.118	0.320	0.118	0.325	0.115	0.315
Conscientiousness (OP.CO)	0.011	0.118	–	–	–	–
Work enjoyment (WbEN)	0.009	0.110	0.009	0.115	0.023	0.162
Agreeableness (OP.AG)	–	–	0.008	−0.104	–	–
Total explained variance (%)	13.8		13.5		13.8	

All the data are significant at < 0.01 (bilateral).

The results also have several theoretical and practical implications. Our study contributes to the body of literature that examines the relationships between workaholism, personality, obsessive beliefs, and entrepreneurial motivation. The practical implications suggest that entrepreneurship support programs could benefit from considering not only entrepreneurial orientation but also other variables such as perfectionism and work enjoyment. This study has not only enriched the field of entrepreneurship and business psychology but also provided insights for future research (Asad et al., 2024; Franczak et al., 2024).

## Limitations and future research

The study presents several limitations that must be considered when interpreting its findings. One of the primary limitations is the complex and multifaceted relationship between the exploratory variables and entrepreneurial motivation. The study acknowledges that not all individuals possessing the related variables will exhibit entrepreneurial motivation and therefore calls for further research to gain a deeper understanding of this relationship. Futhermore, the selected sample represents a limitation as it is predominantly Western and reflects a culture oriented towards competitiveness.

Another area for improvement is the reliance on self-reported data, which may not fully capture external factors such as cultural, social and economic environments. These external factors may also be crucial in shaping an individual’s entrepreneurial motivation. Future research designs should incorporate both internal and external factors to gain a more comprehensive understanding of the drivers behind entrepreneurial motivation.

Additionally, the study only focuses on a limited set of explanatory variables, which may not capture the full range of factors that contribute to entrepreneurial motivation. Therefore, future research should expand the scope of related variables and consider other relevant factors such as family background, educational level, organizational support and previous work experience. Finally, it must be noted that the study’s results may not be universally applicable. The impact of the explanatory variables on entrepreneurial motivation may vary with geographical and cultural differences. Future research should explore the generalizability of the findings across different regions and cultures. This will enable a more nuanced understanding of entrepreneurial motivation.

## Data Availability

The raw data supporting the conclusions of this article will be made available by the authors, without undue reservation.
